# Relevant biomarkers of kidney allograft rejection

**DOI:** 10.25122/jml-2022-0181

**Published:** 2022-11

**Authors:** Luminița Loga, Lucia Dican, Horea Vladi Matei, Ion Mărunțelu, Ileana Constantinescu

**Affiliations:** 1 Clinical Institute of Urology and Renal Transplant, Cluj-Napoca, Romania; 2 Department of Cell and Molecular Biology, Iuliu Haţieganu University of Medicine and Pharmacy, Cluj-Napoca, Romania; 3 Department of Biochemistry, Iuliu Haţieganu University of Medicine and Pharmacy, Cluj-Napoca, Romania; 4 Immunology and Transplant Immunology, Carol Davila University of Medicine and Pharmacy, Bucharest, Romania; 5 Centre of Immunogenetics and Virology, Fundeni Clinical Institute, Bucharest, Romania

**Keywords:** HLA, sensitization, biomarkers, kidney transplant, rejection, CDC – complement-dependent lymphocytotoxicity, dd-cf DNA – Donor-derived cell-free DNA, DNA – deoxyribonucleic acid, HLA – Human leukocyte antigens, LSA – Luminex Single Antigen, miRNA – micro-RNAs, NK – Natural killer, PRA – panel reactive antibody, TGF-β – transforming growth factor-beta

## Abstract

This review focuses on the new relevant biomarkers proposed for the diagnosis of different types of allograft rejections. The immune response against the transplanted tissues can lead to rejection. Kidney allograft rejection occurs when the recipient component's immune system reacts against the donor's cells. MicroRNAs, dd-cf DNA, CD103 markers, CXCR3 chemokine receptor, IP-10, KIR genes, HLA antibodies, the perforin and granzyme B molecules – the constant assessment of all these parameters could prevent acute rejection episodes and kidney injuries. In this way, both immune response and tissue destruction biomarkers are essential for the long-term survival of kidney-transplanted patients. They also contribute to personalizing treatments, precisely personalized immunosuppressive regiments.

## INTRODUCTION

Despite the progress in developing immunosuppressive treatments, graft dysfunction and rejection (acute, chronic) are still common in kidney transplant patients. Nowadays, renal graft function is assessed by determining serum creatinine and urea levels by urinary sediment analysis and renal biopsy. According to Khater and Khauli [1], the biopsy of a kidney graft can help nephrologists identify the immunological and non-immunological causes of renal graft rejection: vascular (renal vein or artery thrombosis), parenchymal (humoral or cellular mediated rejection, recurrent end-stage renal disease), urologic (ureteral edema/obstruction/strictures), collections (abscess/urinoma), tumors (posttransplant lymphoproliferative disorder).

Biopsy is not always accepted by patients and doctors because it is an invasive technique (risk of infections or hematoma) with variable specificity, a high rate of subjectivity findings (dependent on the pathologists' experience), and a lower reproducibility rate [2].

Because none of these markers determine the risk of renal allograft rejection or dysfunction in the early stage, new biomarkers of such measures are needed. In recent decades a number of biomarkers have been proposed that could be used to monitor renal transplant patients. The Food and Drug Administration/National Institutes of Health Biomarker Working Group classify the biomarkers according to the purpose of their study into diagnostic, predictive, prognostic monitoring, and response biomarker [3].

In this review, we mention some of the biomarkers proposed in managing kidney transplanted patients.

### Human leukocyte antigens (HLA)

The major histocompatibility complex (MHC), also known as HLA, plays a significant role in organ transplantation. HLA is a group of linked genes encoding cell surface antigens located on the short arm of chromosome 6 [4], and their location in a defined chromosome area determines inheritance in a block as a haplotype. An individual inherits a haplotype of HLA genes concurrently from each parent, resulting in the individual HLA profile, the group consisting of the two haplotypes being called the genotype. The most determinant factor in renal transplantation is HLA. HLA antigens from the donor cells can induce the activation of the recipient's immune system. This immune response involves both innate and adaptative systems, which will finally affect the function of the kidney allograft [5, 6].

HLA mismatches on HLA-A, -B, and -DRB1 loci are important immunological obstacles in kidney transplantation. Antigens are presented by the HLA molecules, which act as receptors. T cells recognize foreign antigens only when HLA molecules are present. Class I HLA molecules are recognized by cytotoxic T lymphocytes, whereas class II HLA molecules are recognized by T helper lymphocytes [7]. Graft rejection is more likely to occur in the case of donor-patient HLA mismatched. A large number of HLA antigens and the huge number of HLA antigens combinations [8] makes the mission to find a perfect HLA match almost impossible.

### HLA sensitization

HLA antigens are the most important in assessing immunological compatibility between donors and recipients. If they are very different, then the risk of an immune rejection response to the graft is very high, as the recipient's body recognizes the cells of the transplanted organ as foreign. The developments of immunosuppressive therapies have allowed the survival of the grafted organ by combating immunological reactions.

The presence of antibodies against HLA molecules defines HLA sensitization. Anti-HLA antibodies will occur as a result of direct contact with non-self HLA molecules through pregnancy, transfusion, or previous transplantation [9]. The antibodies can also be specific for epitopes shared by HLA molecules of the graft and the host, thus contributing to pathology.

Previous exposures to HLA molecules are associated with a high panel reactive antibody value (PRA%) which estimates a positive crossmatch with the potential donors [10]. Recipients with preformed donor-specific antibodies have the highest risk of graft loss. HLA antibodies can be identified using Luminex Single Antigen (LSA) beads assay available on the X-MAP (Luminex) platform [11]. The assessment of the anti-HLA antibodies specificities is extremely useful for post-transplant monitoring of HLA antibodies to investigate and prevent antibody acute mediate rejection. A potential kidney recipient will spend a longer time on the waiting list for transplantation due to the high risk of different kinds of complications, such as graft rejection and/or side effects of immunosuppressive drugs.

### Natural killer (NK) cells

Natural killer (NK) cells are one of the innate immune system components that modulate the immune response through different factors (such as proinflammatory cytokine) against infections and tumors [11]. Killer cell immunoglobulin-like receptors (members of the immunoglobulin superfamily) control the activity of NK and T cells. Up until now, 14 expressed and 2 pseudo-genes have been identified [12]. The great diversity of killer immunoglobulin receptors (KIR) contributes to the generation of a particularly diverse repertoire of NK cells. NK cells are highly associated with many diseases (especially autoimmune diseases and cancers), graft survival, or the defense against viral infections [12].

To control NK cell actions, activating and inhibitory signals must be harmonized. Because of the high polymorphism rate, the recipient inhibitor's KIR receptor may not be able to identify class I HLA molecules from the donor cells, resulting in NK cell activation against the kidney allograft.

The genes encoding KIR receptors are placed in order, one after another, on chromosome 19q13.4, leading to the identification of two possible haplotypes (A and B) [13, 14]. Compared to haplotype B, which has several combinations of KIR activating and inhibitory genes, including 2DL2, and 2DS1, haplotype A has seven genes: 2DL1, 2DL3, 2DL4, 3DL1, 3DL2, 3DL3-KIR inhibitors, and just one 2DL4 activating gene [15]. A null allele for the 2DL4 gene is present in the population at a frequency of roughly 84 percent [14]. As a result, certain people who have the haplotype A allele may not express the KIR-activating receptor. In the Caucasian population, the two haplotypes are distributed relatively equally. KIR receptor ligands HLA-C1, C2, and HLA-B are well-defined. Individuals having a particular KIR receptor may lack the corresponding HLA ligand, the KIR receptor being thus inoperable [14, 15].

KIR2DS1 and KIR2DL1 bind to the HLA-C2 group, while a set of HLA-Cw molecules containing Lysine in position 80 are recognized by KIR2DL2/3 and KIR2DS2. The activating KIR3DL1 and 3DS1 bind to HLA-Bw4 [16].

### Compatibility testing between donor and recipient and clinical utility

Better HLA compatibility between recipients and their related/unrelated donors is essential for allograft survival. The serological HLA typing technique was the first method used to determine the antigens. The method was developed by Terasaki and McClelland in 1964, who created a method based on complement-dependent lymphocytotoxicity (CDC).

The typing of HLA antigens can be done through serological techniques and molecular techniques (SSP-Sequence Specific Primer, SSO-Sequence Specific Oligohybridization, Sequencing Based Typing, Next-Generation Sequencing, Microarray). Molecular typing is superior to serological methods for determining allele group subtypes [17–19]. Routinely we genotype class I HLA (HLA-A, -B, and HLA-C) and class II HLA (HLA-DQB1, -DPB1, and -DRB1). Microarray technology can discriminate between deoxyribonucleic acid (DNA) sequences that may vary only by one nucleotide. DNA hybridization is based on the principle of complementary linkage of single-strand nucleic sequences on a microarray (or microchip); known sequences are attached to designated locations (points) on a solid surface, such as glass, using robotic scoring [20]. Two types of microarray DNA are commonly used: DNA genomic matrix, with complementary DNA sequences of 500–5000 base pairs that are immobilized, each representing a gene or specific chromosome fragment, and oligo-matrices, which are microchips with shorter DNA sequences of 20–100 base pairs fixed as targets. Unknown solution sequences called probes will bind to immobilized complementary targets. The same principle applies to other purposes and probes, such as proteins, glycans etc. For HLA typing, microarray technology will have to prove its efficiency and performance because the sequence is a competitive competitor in terms of sensitivity, specificity, and decipherment of new alleles [20–22].

### Biological markers

Biomarkers can be used to diagnose humoral or cell-mediated rejection, predict the allograft outcome, and immediately detect an injury. Newly discovered biomarkers are used to identify patients who are tolerant or almost tolerant and detect chronic renal disease in its early stages and help separate it from different forms of chronic homograft nephropathies with immune responses as well as true chronic rejection [23]. This reality allows to soundly scale back or withdraw the immunological disorder medical care [23].

### Genomics, proteomics, metabolomics, and transplantation

In addition to actual parameters used in many transplant centers, biomarkers can also be used in clinical decisions [24]. According to Li et al., the levels of perforin and granzyme B can be quantified in the kidney transplant patient's urine to screen for acute rejection [25–27].

In the last decades, other potential biomarkers were proposed, markers that can provide additional information related to the risk of acute rejection occurrences, such as CD103 markers [24, 25], the chemokine receptor CXCR3, or the protein IP-10 [28, 29].

### Donor-derived cell-free DNA (dd-cf DNA) and transplantation

A potential early indicator for the injury and loss of allograft is dd-cf DNA, which is donor-derived DNA circulating in the blood or urine of transplanted patients [30].

Since there is no active degradation of the allograft, the normal level of dd-cf DNA represents less than 1% of unnucleated cells. The test does not need any more previous genotyping of the donor or recipient and determines the percentage of total cell-free DNA utilizing targeted amplification and sequencing of single-nucleotide polymorphisms.

Changes that occur right after transplantation are linked to dd-cf DNA. Among kidney transplantation recipients, dd-cf DNA levels are elevated during acute rejection time and decline within the following three months, allowing real-time treatment of acute rejection response monitoring [31]. Thus, dd-cf DNA can be used to identify acute rejection or to assess recovery from acute rejection and improve the long-term monitoring of kidney transplant recipients [27, 31–33].

### MICRO-RNAs (miRNA) and transplantation

miRNAs are often encoded in introns and between genes. They prevent protein translation by binding to the ribosome-binding sites of the messenger RNA. miRNAs can be detected in plasma, urine, and intracellular presences and are associated with many immunological factors that participate in acute or chronic rejection mechanisms. Among these factors, the transforming growth factor-beta (TGF-β) causes fibrosis [34], although it is an inhibitor of many inflammatory pathways. MiRNAs that negatively regulate the TGF-β gene transcriptions are miRNA-548d, miRNA-203, and miRNA-146a [35].

The absence or possibly suppression of the TGF-β released in inflammation and rejection processes with mi-RNAs may cause the balance to shift in the direction of the positive regulators of inflammation and lead to rapid rejection response and graft loss [36]. However, the blockade of mi-RNAs that inhibit the TGF-β increase also leads to an uncontrolled release of TGF-β, increased fibrosis, and complete loss of function [36]. Indeed, TGF-β is associated with certain miRNAs in kidney transplants [36]. Rejection of a kidney transplant can take four different forms such as hyper-acute rejection (extremely rare given the development of antibody detections and treatments [37] and the performance of crossmatch test before the transplant procedure), acute renal rejection (which may appear in the first years after the transplant procedure) and chronic rejection (occurs years after transplant because of both nonimmunological and immunological causes) [38]. MicroRNAs are endogenous ribonucleic acid molecules vital for a replacement step in regulating the organic phenomenon [39]. microRNAs are seen in body fluids in high quantity in case of excretory organ injury (for instance, in nephropathies or allograft failure) [40–42].

Decreased glomerular filtration rate and hypertension are seen in patients who experience acute or chronic rejection [38, 43]. Validation studies and assay standardization for the new biomarkers are needed even if biomarker validations are difficult to be performed because of inter-laboratory and inter-platform variability [39, 40, 42].

## CONCLUSIONS

Our paper revealed multiple arguments favoring a new generation of biomarkers that highly improve clinical follow-up and patient care after kidney transplantation. MicroRNAs, dd-cf DNA, CD103 markers, CXCR3 chemokine receptor, IP-10, KIR genes, HLA antibodies, the perforin and granzyme B molecules – the constant assessment of all these parameters could prevent acute rejection episodes and kidney injuries. In this way, both immune response and tissue destruction biomarkers are essential for the long-term survival of kidney-transplanted patients. They also contribute to personalizing treatments, specifically personalized immunosuppressive regiments.
